# Supplementary material for: Activity of a Recombinant Chitinase of the Atta sexdens Ant on Different Forms of Chitin and Its Fungicidal Effect against Lasiodiplodia theobromae
Source: Polymers (Basel). 2024 Feb 15;16(4):529. doi: 10.3390/polym16040529 (PMC10892911; doi:10.3390/polym16040529)
Supplement: Supplementary file 1 [file polymers-16-00529-s001.zip › polymers-2871800-supplementary.pdf]

Activity of a Recombinant Chitinase of the *Atta sexdens* Ant on Different Forms of the Chitin, and Its Fungicidal Effect Against *Lasiodiplodia theobromae*

Katia Celina Santos Correa[a], William Marcondes Facchinatto[b], Filipe Biagioni Habitzreuter[c], Gabriel Henrique Ribeiro[d], Lucas Gomes Rodrigues[a], Kelli Cristina Micocci[a], Sérgio Paulo Campana-Filho[c], Luiz Alberto Colnago[d], Dulce Helena Ferreira Souza[a]

[a] Department of Chemistry, Federal University of Sao Carlos (UFSCar), Rd. Washington Luis, km 235, CEP – 13565-905, Sao Carlos, SP, Brasil.

[b] Aveiro Institute of Materials (CICECO), Department of Chemistry, University of Aveiro, R. Santiago, CP – 3810-193, Aveiro, Portugal.

[c] Sao Carlos Institute of Chemistry, University of Sao Paulo (IQSC-USP), Av. Trabalhador Sao-carlense 400, CEP – 13560-590, Sao Carlos, SP, Brasil.

[d] Brazilian Agricultural Research Corporation (Embrapa Instrumentation), R. XV de Novembro 1452, CEP – 13560-970, São Carlos, SP, Brasil.

*Corresponding author:

Dulce Helena F. Souza

E-mail address: dulce@ufscar.br

Phone number: +55 16 3351 8074

Department of Chemistry, Federal University of São Carlos

Rd. Washington Luis, km 235, CEP – 13565-905

São Carlos - SP, Brasil

Summary

Captions	Page
**Figure S1.** GlcNAc chemical structure.	**S2**
**Figure S2.** COZY ^1H-^1H NMR spectrum for GlcNAc.	**S4**
**Figure S3.** Enzymatic activity in the presence of colloidal α-chitin substrate at 28 °C.	**S5**

Caption	Page
**Table S1.** Assignments of the ^1H NMR spectra in D_2O for the N-acetyl-d-glucosamine compound	**S3**

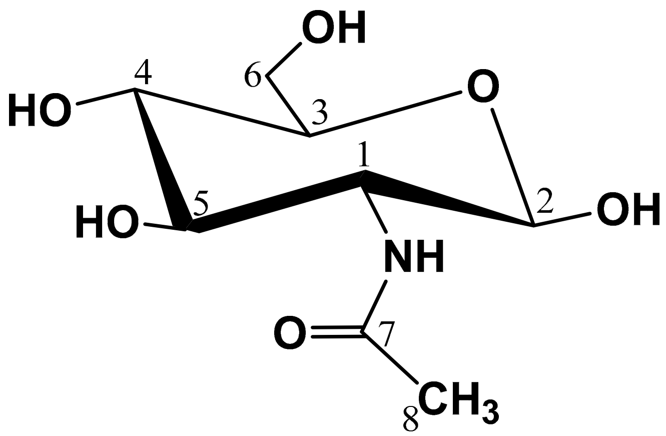

Figure 1S. GlcNAc chemical structure.

Table 1S. Assignments of the ^{1}H NMR spectra in D_2O for the N-acetyl-d-glucosamine compound. Chemical shifts (ppm), multiplicity, and coupling constants (Hz)

**No.**	**^{1}H**
1’	3.88 (m)
2’	5.19 d (2.8 Hz)
3’	3.40 – 3.95 (m)
4’	3.40 – 3.59 (m)
5’	3.40 – 3.95 (m)
6’	3.40 – 3.95 (m)
NH	8.09 d (8.0 Hz)
7’	-
8’	2.03 s

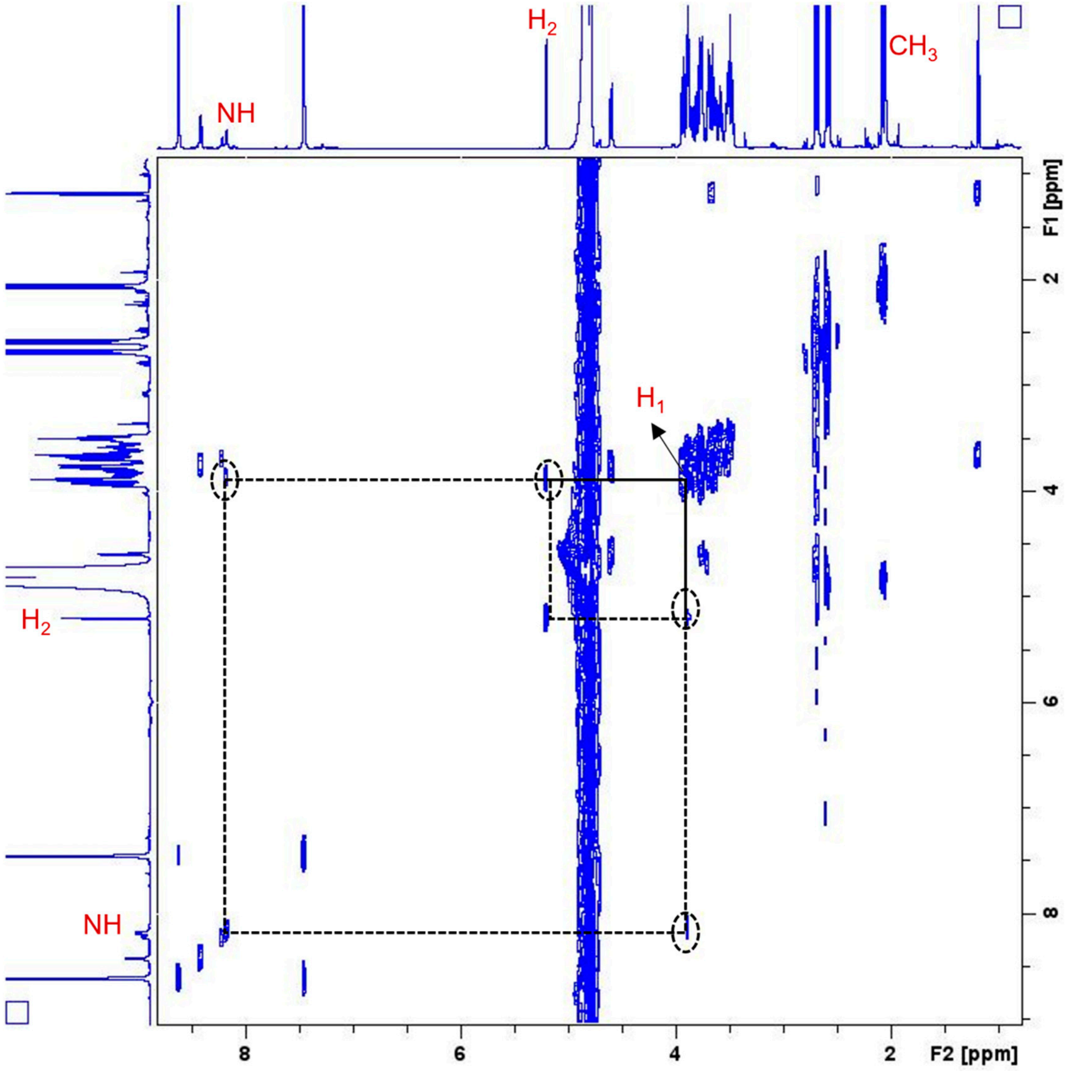

Figure S2. COZY ^1H-^1H NMR spectrum for GlcNAc. Signals of the N-acetil-d-glucosamina hydrolyzed product are highlighted.

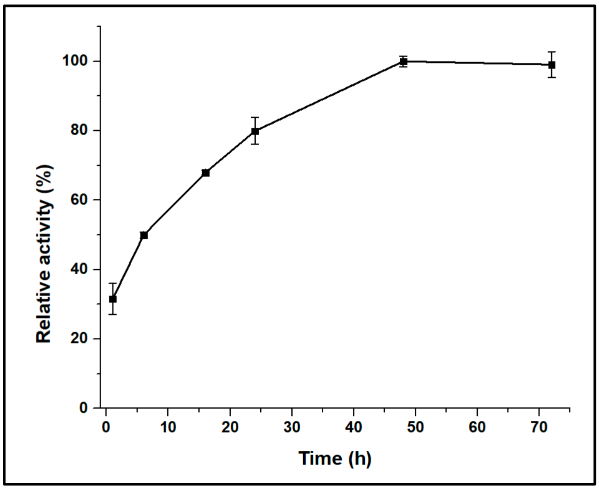

Figure S3. Enzymatic activity in the presence of colloidal α-chitin substrate at 28 °C.
